# Demyelinating disease masquerading as a surgical problem: a case series

**DOI:** 10.4076/1752-1947-3-7407

**Published:** 2009-08-05

**Authors:** Saufi M Awang, Nayan M Saiful, Mohan Madhavan, Jafri Abdullah, John K Tharakan

**Affiliations:** 1 Department of Neurosciences, School of Medical Sciences, Universiti Sains Malaysia, Kelatan, Malaysia; 2 Department of Pathology, School of Medical Sciences, Universiti Sains Malaysia, Kelatan, Malaysia

## Abstract

**Introduction:**

We report three cases of demyelinating disease with tumor-like presentation. This information is particularly important to both neurosurgeons and neurologists who should be aware that inflammatory demyelinating diseases can present as a mass lesion, which is indistinguishable from a tumor, both clinically and radiologically, especially when there is no evidence of temporal dissemination of this disease.

**Case presentation:**

The first patient was a 42-year-old Malay woman who developed subacute onset of progressive quadriparesis with urinary incontinence. Magnetic resonance imaging of her spine showed an intramedullary lesion at the C5-C7 level. She was operated on and biopsy was suggestive of a demyelinating disease. Retrospective history discovered two episodes of acute onset of neurological deficits with partial recovery and magnetic resonance imaging of her brain revealed demyelinating plaques in the centrum semiovale.

The second patient was a 16-year-old Malay boy who presented with symptoms of raised intracranial pressure. A computed tomography brain scan revealed obstructive hydrocephalus with a lesion adjacent to the fourth ventricle. An external ventricular drainage was inserted. Subsequently, a stereotactic biopsy was taken and histopathology was reported as demyelination. Retrospective history revealed similar episodes with full recovery in between episodes.

The third case was a 28-year-old Malay man who presented with acute bilateral visual loss and confusion. Magnetic resonance imaging of his brain showed a large mass lesion in the right temporoparietal region. Biopsy was consistent with demyelinating disease. Reexamination of the patient revealed bilateral papillitis and not papilledema. Visual evoked potential was prolonged bilaterally. In all three cases, lumbar puncture for cerebrospinal fluid study was not carried out due to lack of patient consent.

**Conclusions:**

These cases illustrate the importance of considering a demyelinating disease in the differential diagnosis of a mass lesion. Critical analyses of clinical presentations coupled with good physical examination are vital in assisting clinicians to reach the correct diagnosis.

## Introduction

Inflammatory demyelinating diseases can present as a mass lesion, which is indistinguishable from a tumor, both clinically and radiologically, especially when there is no evidence of temporal dissemination of this disease, posing a challenge to the clinician [1]. In countries where the prevalence of multiple sclerosis (MS) and acute disseminated encephalomyelitis (ADEM) is low, the index of clinical suspicion will be very low and this can lead to misdiagnosis. We report three cases of demyelinating disease, all of which presented as a mass lesion mimicking a tumor.

## Case presentation

### Case 1

**Figure 1:**
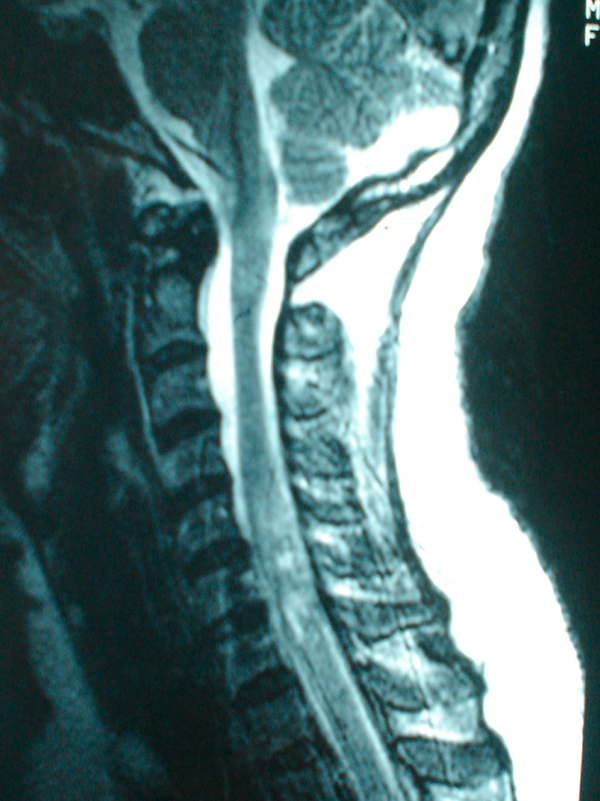
**Sagittal T2-weighted image of the cervical spine showing an intramedullary lesion at the C5-C7 spinal vertebrae level**.

A 42-year-old Malay woman was referred to us for the surgical management of an intramedullary tumor of the spinal cord in July 2002. Her symptoms started in April 2002 as subacute onset progressive quadriparesis and by the time of admission, she had developed urinary incontinence and had become bedridden. A magnetic resonance imaging (MRI) scan of the spine was reported to show an intramedullary tumor at the C1-C5 level (Figure 1). Decompressive laminectomy and biopsy of the lesion were carried out in July 2002. The histological features were consistent with demyelinating disease. On review of her medical history, she reported two previous episodes of acute onset of neurological deficits with partial recovery in October 1998 and March 2002 presenting as mild paraparesis with bladder involvement over a period of 2 weeks with a complete return to the previous baseline. Physical examination showed a bilateral pale disc with normal visual acuity, quadriparesis with brisk reflexes and upgoing plantar responses and sensory loss at T12-L1. Routine blood investigations, serology for syphilis, hepatitis and HIV, and screening for autoimmune antibodies were all negative. Visual evoked potential (VEP) was slightly prolonged in the right eye. Brainstem auditory evoked potential (BAEP) was within normal limits. An MRI scan of her brain revealed demyelinating plaques in the centrum semiovale. Based on additional information on remitting and relapsing neurological deficits involving the optic nerve, the spinal cord and the brain, the diagnosis was revised to MS. She was treated with interferon beta 1a (Rebif) three times weekly. At follow-up one year later, she reported a significant improvement in her functional capabilities. She was able to walk, had regained bladder control and could perform activities of daily living on her own.

### Case 2

**Figure 2:**
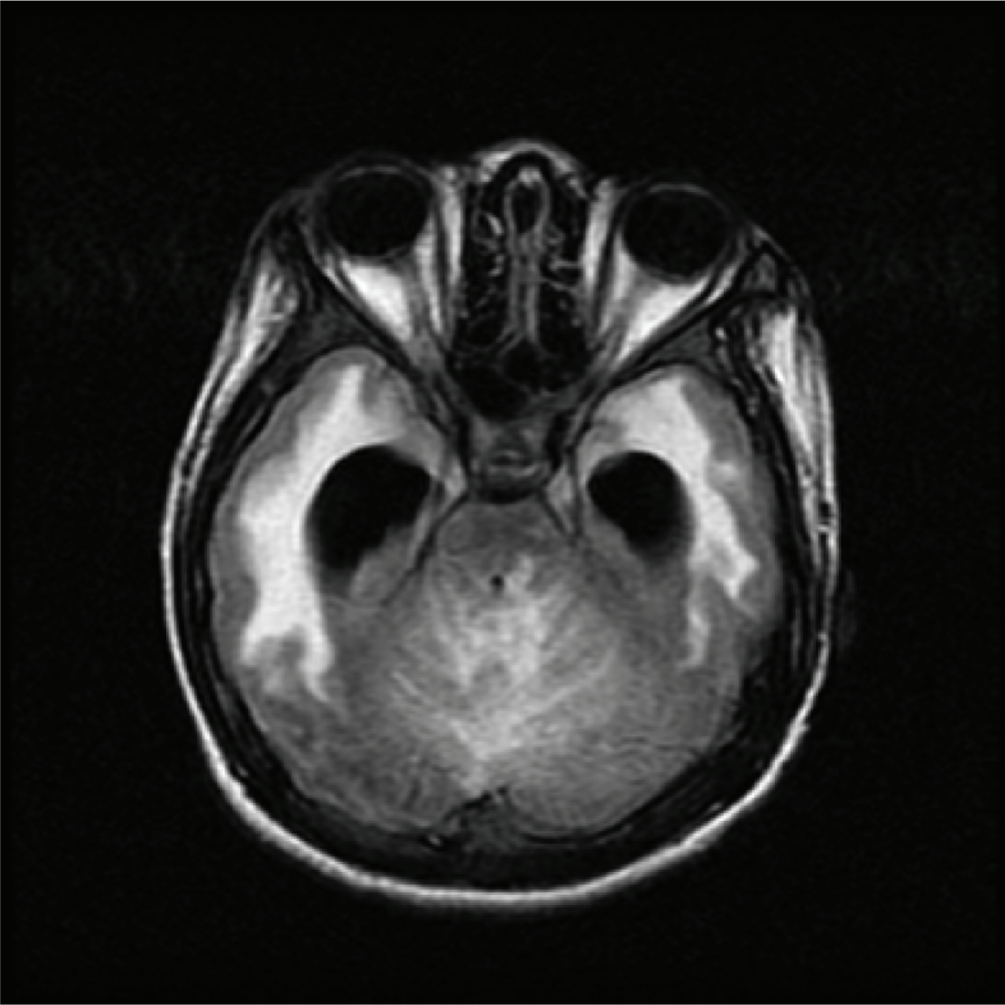
**Axial fluid-attenuated inversion recovery sequence showing multiple lesions in the brainstem and cerebellum**.

A 16-year-old Malay boy was admitted to our hospital in December 2003 for surgical management of increased intracranial pressure. He underwent external ventricular drainage and stereotactic biopsy. The histological report was consistent with demyelination. He had recurrent episodes of acute onset severe headache and vomiting with a mild unsteady gait, first in August 2003, then in September 2003 and the last in October 2003 leading to hospital admission. In the first two occasions, CT and MRI scans revealed obstructive hydrocephalus with a lesion, enhanced with contrast in the region of the fourth ventricle. He was treated with steroids since he refused surgery and recovered fully in 1 to 2 weeks. He was well until early October 2003 when he had recurrent headache and subacute onset of a severe unsteady gait. Because of this, he became bedridden. The symptoms continued and in November 2003, he complained of blurring of vision and lost his sight 3 weeks later. The examination revealed bilateral optic neuritis with multiple left cranial nerve palsy (IX, X and XII), bilateral pyramidal tract signs (more prominent on the right side) and cerebellar signs. Autoimmune antibodies and serology screening for HIV, syphilis and hepatitis B and C were negative. VEP was absent in both eyes. BAEP was suggestive of bilateral auditory pathway dysfunction. The MRI brain scan showed multiple small lesions in the thalamus, centrum semiovale and brainstem and an ill-defined lesion in the cerebellum which appeared to be hypointense on T1-weighted images and hyperintense on T2-weighted images, not suppressed by fluid-attenuated inversion recovery (FLAIR), and enhanced with contrast (Figure 2). In view of the remitting and relapsing course of the illness, involving multiple sites in the central nervous system - optic nerve, brainstem, cerebellum and cortex - his diagnosis was revised to MS. The cause of his hydrocephalus was a strategically located demyelinating plaque causing compression of the fourth ventricle. He was treated with intravenous methylprednisolone for five days and tapering oral prednisolone for one month and showed some improvement to the extent that he was able to perceive light and sit up without support.

### Case 3

**Figure 3:**
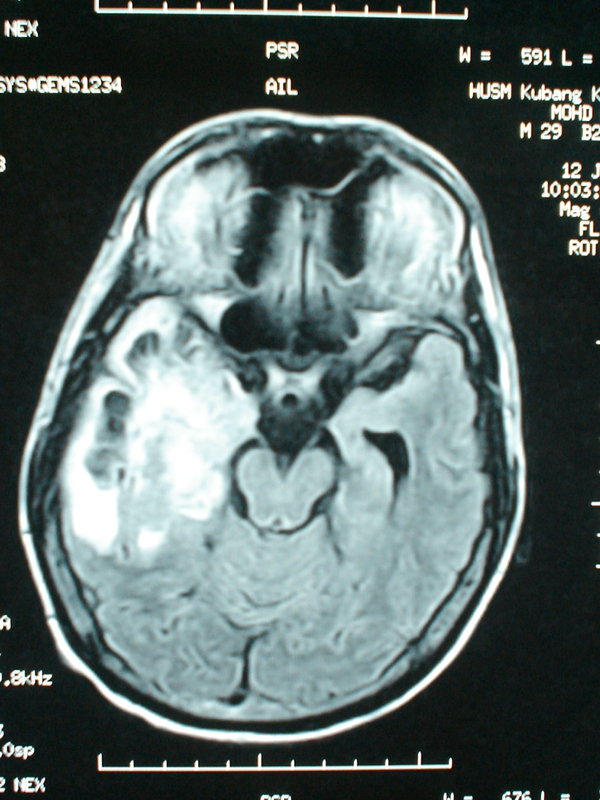
**Axial fluid-attenuated inversion recovery sequence showing a large mass lesion located in the right temporal region**.

In May 2003, a 28-year-old man, known to sniff petrol, was referred to us for further management of a suspected brain tumor. His symptoms started as acute onset severe bilateral visual loss and confused behavior of two weeks' duration. Initial neurological examination showed reduced visual acuity to light perception only, symmetrical reactive pupils, bilateral swollen optic disc and left-sided pyramidal signs. The MRI brain scan on admission was reported to show a large mass lesion in the right temporoparietal region which was hypointense on T1-weighted images and hyperintense on T2-weighted images with heterogeneous enhancement after gadolinium and not suppressed on FLAIR sequence (Figure 3). A stereotactic biopsy was carried out; histology was consistent with demyelinating disease. Re-examination of this patient revealed that his pupillary reflex was sluggish with visual acuity reduced to perception of light only and he had bilateral papillitis and not papilledema. Serology screening for HIV, syphilis, and hepatitis B and C were negative and BAEP was normal. VEP was grossly prolonged bilaterally. Given the abrupt onset of symptoms, bilateral papillitis and a large demyelinating lesion in the brain without any evidence of dissemination in time, his diagnosis was revised to acute disseminated encephalomyelitis. He was started on intravenous methylprednisolone 1 g daily and showed a dramatic improvement after 5 days of steroid. He was discharged with a tapering dose of oral prednisolone. At follow-up after three months, he was able to walk without support, had normal mentation and improved visual acuity to finger counting.

## Discussion

These three cases are examples of inflammatory demyelinating disease masquerading as lesions requiring neurosurgical intervention. Neurosurgeons should be aware of this and carefully review the patient's clinical history when making a diagnosis. In the first patient, a thorough and proper clinical evaluation should have identified the evidence of demyelinating disease In the second patient, a rare complication of an uncommon disease in this region misled the diagnosis. The third patient had an atypical imaging finding of a relatively uncommon disease which led to the initial wrong diagnosis. In all three instances, a very low index of suspicion for demyelinating disease, except for one case, contributed to unnecessary surgical interventions.

Many criteria have been proposed to guide clinicians in making a diagnosis of MS but the current guidelines widely followed in clinical practice are the ones proposed by McDonald *et al.*[2]. The criteria comprise the combination of clinical presentation, that is to say, remitting and relapsing course of illness, with the number of attacks, findings on neurological examination, evidence from MRI scans, and supportive ancillary testing such as cerebrospinal fluid evaluation for IgG index and oligoclonal bands, and visual evoked potential tests. In typical MS, the MRI lesions will appear hyperintense on T1 images enhanced with gadolinium and on T2-weighted images including FLAIR sequence [3,4]. Usually, the lesions are disseminated in white matter and a mass effect is not seen [5].

The first patient had spinal MS with a tumor-like presentation, clinically and radiologically with subacute onset progressive myelopathy and MRI evidence indicative of intramedullary lesion. The spinal MRI features of Asian patients with MS are slightly different compared with Western patients with MS in terms of the size and length of lesions and association with cord swelling. According to Chong *et al.*[6], the average size of spinal cord lesions in Asian patients is 3.6 vertebral body segments, larger than in Western patients. Lee *et al.*[7] found that 9 out of 212 patients who underwent surgery for intramedullary spinal cord tumors had non-neoplastic lesions, of which half of these lesions were reported as demyelination. However, given the remitting and relapsing course of the illness in our patient, inflammatory demyelinating diseases should be included in the differential diagnosis.

The second case described here demonstrates that inflammatory demyelinating disease can present as a surgical emergency if a strategically located lesion causes obstructive hydrocephalus. Butler and Gilligan [3] reported a case of obstructive hydrocephalus due to an inflammatory demyelinating plaque causing a mass effect in the brainstem. In addition, this case also demonstrates that a thorough clinical history and good physical examination will assist the clinician in making a correct diagnosis and avoiding unnecessary biopsies. In the setting of acute hydrocephalus with complete resolution as confirmed on imaging after steroid treatment, plus the acuteness of the blindness which was disproportionate to the degree of hydrocephalus suggesting papillitis rather than papilledema, this should have raised the index of suspicion toward the diagnosis of demyelinating disease.

The third case described here highlights the fact that ADEM can present as a solitary mass lesion. ADEM is an acute demyelinating condition affecting the brain and spinal cord and usually occurs following an infection or vaccination. More importantly, it is a monophasic disease. Typically, the MRI findings of ADEM include lesions of the same age and no new lesions after the initial onset. The corpus callosum is usually not involved [8]. Singh *et al.*[9] retrospectively studied 13 patients with solitary hemispheric lesions of whom 12 patients were diagnosed with ADEM based on MRI features and clinical parameters. In the majority of these cases, brain biopsy was not indicated although Comi [10] estimated that biopsy is fundamental in 20-30% of cases. The other important point to stress here is the confusion between papillitis and papilledema. Usually papillitis is unilateral instead of bilateral, with less elevation of the nerve head and sluggish papillary response compared with papilledema [11]. These findings can be used to guide the clinicians in distinguishing between the two.

Another issue that needs to be highlighted is regarding neuromyelitis optica. There is controversy as to whether the optico-spinal variant of Asian MS is a clinical subtype of MS or a distinct entity of neuromyelitis optica. Recent reports of detection of neuromyelitis optica antibodies in the serum of these patients favor the latter condition. Thus, the new diagnostic criteria for neuromyelitis optica are based on clinical, MRI and neuromyelitis optica antibody results [12].

## Conclusion

We conclude that demyelinating disease can occasionally present as a tumor and only a proper and thorough history coupled with good physical examination and MRI scanning will help clinicians reach the correct diagnosis.

## Abbreviations

ADEM: acute disseminated encephalomyelitis; BAEP: brainstem auditory evoked potential; CT: computed tomography; FLAIR: fluid-attenuated inversion recovery; MRI: magnetic resonance imaging; MS: multiple sclerosis; VEP: visual evoked potential.

## Consent

Written informed consent was obtained from the patients for publication of this case report and any accompanying images. Copies of the written consent are available for review by the Editor-in-Chief of this journal.

## Competing interests

The authors declare that they have no competing interests.

## Authors' contributions

SMA analyzed the cases, collected the images and formulated the manuscript. NMS completed the literature review. MM was the pathologist reporting the histopathology slides. JA carried out the literature review and corrected the manuscript. JKT reviewed and corrected the manuscript. All authors read and approved the final manuscript.
